# Catheter manipulation analysis for objective performance and technical skills assessment in transcatheter aortic valve implantation

**DOI:** 10.1007/s11548-016-1391-6

**Published:** 2016-04-12

**Authors:** Evangelos B. Mazomenos, Ping-Lin Chang, Radoslaw A. Rippel, Alexander Rolls, David J. Hawkes, Colin D. Bicknell, Adrien Desjardins, Celia V. Riga, Danail Stoyanov

**Affiliations:** Centre for Medical Image Computing and Department of Computer Science, University College London, London, WC1E 6BT UK; Division of Surgery, Department of Surgery and Cancer, Imperial College London, London, SW7 2AZ UK; Department of Medical Physics and Bioengineering, University College London, London, WC1E 6BT UK

**Keywords:** Objective skills assessment, Catheter motion analysis, Endovascular robotics

## Abstract

**Purpose:**

Transcatheter aortic valve implantation (TAVI) demands precise and efficient handling of surgical instruments within the confines of the aortic anatomy. Operational performance and dexterous skills are critical for patient safety, and objective methods are assessed with a number of manipulation features, derived from the kinematic analysis of the catheter/guidewire in fluoroscopy video sequences.

**Methods:**

A silicon phantom model of a type I aortic arch was used for this study. Twelve endovascular surgeons, divided into two experience groups, experts ($n=6$) and novices ($n=6$), performed cannulation of the aorta, representative of valve placement in TAVI. Each participant completed two TAVI experiments, one with conventional catheters and one with the Magellan robotic platform. Video sequences of the fluoroscopic monitor were recorded for procedural processing. A semi-automated tracking software provided the 2D coordinates of the catheter/guidewire tip. In addition, the aorta phantom was segmented in the videos and the shape of the entire catheter was manually annotated in a subset of the available video frames using crowdsourcing. The TAVI procedure was divided into two stages, and various metrics, representative of the catheter’s overall navigation as well as its relative movement to the vessel wall, were developed.

**Results:**

Experts consistently exhibited lower values of procedure time and dimensionless jerk, and higher average speed and acceleration than novices. Robotic navigation resulted in increased average distance to the vessel wall in both groups, a surrogate measure of safety and reduced risk of embolisation. Discrimination of experience level and types of equipment was achieved with the generated motion features and established clustering algorithms.

**Conclusions:**

Evaluation of surgical skills is possible through the analysis of the catheter/guidewire motion pattern. The use of robotic endovascular platforms seems to enable more precise and controlled catheter navigation.

## Introduction

Minimally invasive (MI) endovascular interventions are nowadays the favoured option for treating a number of vascular diseases, particularly in high-risk patients with comorbidities that are deemed unsuitable for open heart surgery [7]. Transcatheter aortic valve implantation (TAVI) is a MI procedure for replacing a malfunctioning stenosed aortic valve, where a catheter is percutaneously inserted in the vasculature and navigated through the aorta in order to deploy the artificial valve. TAVI operations offer the advantage of shorter recovery times for patients, since the operational trauma is minimal. However, TAVI is a complex procedure that requires surgeons to navigate steerable instruments in the fragile, often highly sclerotic vascular system, under friction and the presence of calcium deposits. This, combined with the restricted visualisation of the operating environment, raises the risk of embolisation and tissue damage (e.g. vessel rupture) due to intra-operative errors. Modern endovascular surgeons must possess a high level of manual dexterous ability as well as be able to operate under significant physical and mental load.

Performance evaluation is a fundamental element of outcome-driven medicine and healthcare delivery, yet objective methods and metrics for developing training programmes and evaluating surgical performance and workflow are not fully developed or in widespread use. Traditionally, surgical expertise is evaluated with written and oral examinations, procedural and case logs, as well as expert monitoring during exercises and studies on cadavers, animals, phantom models or virtual simulators [16]. A more objective approach is the use of standardised, structured grading scales (GRS) or checklists which again require expert observation and manual scoring of the trainee either in real time or through reviewing video recordings. Although subjective to some extent, these methods are the current gold standard for performance evaluation. The problem in their application arises due to inefficiency issues since they are laborious and time-consuming. In addition, supervised assessment is inherently subjective to a degree and introduces bias which makes difficult the global standardisation of an acquired level of technical expertise [9].

In recent years, research efforts have focused on developing methods for evaluating surgical skills that are more objective and can complement traditional expert assessment [10, 20, 23]. Empirical evidence suggests that the level of technical expertise is represented by the proper handling of surgical equipment in such a way that is efficient and minimises the risk of errors occurring [3, 15]. In the endovascular domain, the previous work attempted to objectively characterise the skill level of surgeons through analysing the kinematics of the catheter and other surgical tools and extracting features and metrics. Rolls et. al. utilised software to track the position of the catheter’s tip in experiments with the Procedicus VIST simulator. In results from 21 participants of varying experience, the total path length correlated well with manually scored GRS [21]. Additional metrics from catheter tip tracking data that have been identified as potential indicators of endovascular skills are non-dimensional jerk, spectral arc length, number of sub-movements and average sub-movement duration [4, 5]. Other type of information that has been investigated is the force and torque loads exerted on the catheter by endovascular operators. From experiments with a robotic system, it was deduced that the force/torque loads that were exerted on the catheter by experienced and inexperienced subjects were different. Five features median speed, the mean value of displacement, push force, torque and the number of twists demonstrated correlation with surgical skills [12]. Motion and contact force information were then used for training hidden Markov models (HMM) for classifying cannulation tasks executed from surgeons of varying experience [13]. Recent work on modelling and tracking the entire shape of catheters in fluoroscopic video sequences will lead to a plethora of new kinematic information that may prove extremely useful for discriminating surgical performance [2].

**Fig. 1:**
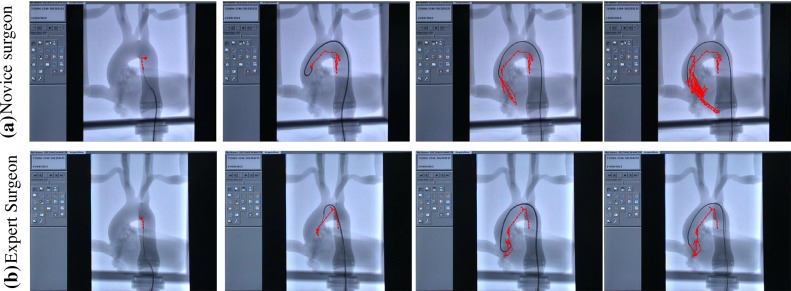
Sequential tracks of a catheter/guidewire tip in different times during the cannulation of an aorta phantom model: **a** a novice surgeon; **b** an expert. As the catheter progresses in the aortic arch, we note the more efficient manipulation of the expert resulting in smoother trajectory

A potential solution for the intricate challenges in TAVI could be the use of robotically controlled surgical systems. Interest in the design and application of robotics for endovascular operations is gradually increasing, with the previous work evaluating potential performance benefits from the use of robotic systems [11]. Advantages include shorter training periods, decreased cognitive load, enhanced precision and catheter manoeuvrability which translates to increased safety and lower risk of vascular damage, compared to conventional catheters [17, 18]. Subsequently, there has been growing interest in the clinical evaluation of robotic systems for TAVI operations [14, 19].

Virtually all established endovascular GRS and checklists contain criteria, albeit their description might differ slightly that evaluate three key technical attributes. These can be summarised as: (1) avoiding vessel wall damage with precise catheter manipulation, especially near delicate or sclerotic areas; (2) smooth catheter navigation in a timely fashion without unnecessary movements; (3) efficient use of the imaging modalities and contrast agents. Focusing on (1) and (2), we hypothesise that potential quantitative measures extracted from analysing medical images and tracking the instruments trajectory and combined appropriately will contain the necessary information to discriminate levels of surgical experience.

Figure 1 illustrates sequential tracks of the tool tip during the cannulation of the aortic arch, from a novice (Fig. 1a) and an expert surgeon (Fig. 1b). We observe that the expert surgeon manipulates the tool in a more efficient, economical way than the novice, resulting in smoother trajectory. We use this information to calculate a number of kinematic features (e.g. speed, acceleration, jerk) for our investigation. Furthermore, we introduce the distance of the tip to the vessel wall as a metric to evaluate the safe navigation of the catheter with respect to adjacent vascular tissue. Statistical analysis results are indicative of the experts developed dexterity and technical competence as represented by lower median values of procedure time and dimensionless jerk, and higher average speed and acceleration than novices. Ultimately, we employed the features that demonstrated statistical significance as inputs in two clustering algorithms (k-means, expectation-maximisation). Our objective was to categorise the participants according to their experience level and the different types of surgical equipment in executions from the same group. The obtained level of accuracy was 83–91 %, for experience group classification and 75–91 % for equipment classification.

## Methods

### Experimental set-up

**Fig. 2:**
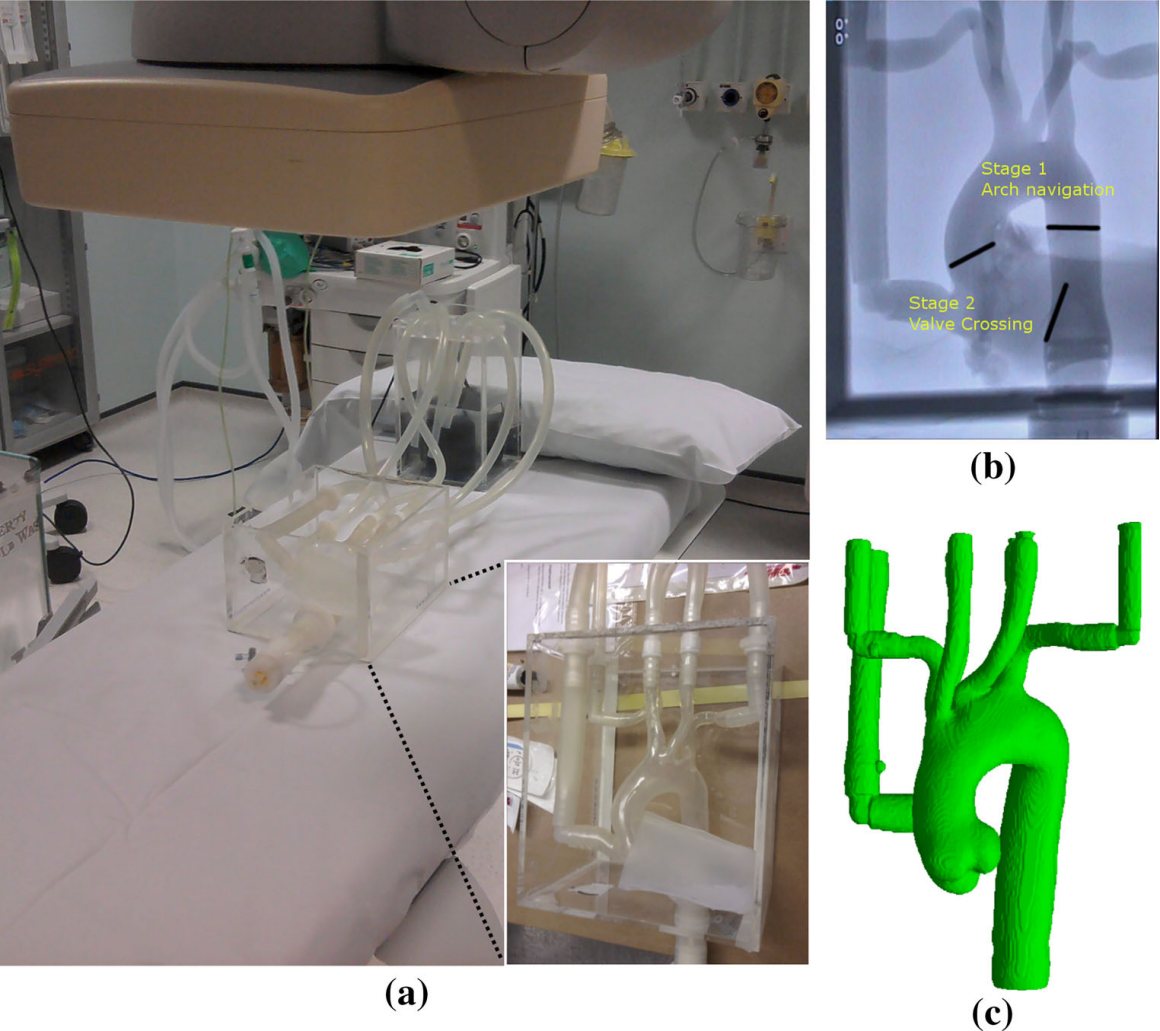
**a** Experimental testbed; **b** boundaries of the two TAVI stages; **c** 3D rendering of the type I aorta phantom

A silicon aorta model (Elastrat Sàrl, Geneva, Switzerland), representing a type I (i.e. the arch vessels arise from the outer curvature of the arch in the same horizontal plane) aortic arch and constructed from CT recordings in humans was used in our experimentation. The experimental testbed and model are illustrated in Fig. 2a. A detailed illustration of the geometry of the aortic arch is provided in Fig. 2c which shows a 3D rendering of the phantom. The opening of the aortic valve was reduced to 0.6 cm$^{\text {2}}$ in such a way so as to resemble stenosis and a morbidity of the valve. The left ventricle was modelled with silicon sheets and attached to the proximal end of the arch. The phantom model was perfused by a water and glycerol solution, circulated via a pump in order to emulate blood circulation.

TAVI procedures were divided into two stages; the first stage was navigation of the catheter/guidewire through the aortic arch, defined as the advancement from the descending aorta (at the point marked by the most proximal part of the left ventricle) into approximately 2 cm proximal to the aortic valve. The second part involved the crossing of the aortic valve and was considered as the advancement of the catheter/guidewire from the 2 cm point proximal to the aortic valve, into the left ventricle. Approximate positions that define the two TAVI stages are depicted in Fig. 2b. These are the initial stages of a real TAVI operation, and given that experimentation takes place on a phantom, completion times are expected to be significantly smaller than in the operating room during real TAVI procedures. However, the navigation of the catheter through the descending aorta, over the arch and into the left ventricle is a challenging cannulation task which requires refined manipulation skills. Subsequently, we anticipate the catheter manipulation characteristics of the two groups to be visible even in the initial stages of a TAVI procedure.

Conventional surgical instruments included typical catheters—5Fr pigtail (Cordis Corporation, Warren NJ, USA) and AL1 guide catheter (Boston Scientific, Natick, MA, USA), guidewires—0.032 in J-wire (Boston Scientific), 0.018 in Glidewire^®^ (Terumo Medical Corp, Somerset, NJ, USA), 0.035 in super-stiff wire (Boston Scientific) and medical balloons, 22-mm Tyshak balloon^®^ (B. Braun Medical Inc, Bethlehem, PA, USA). Fluoroscopic imaging was employed for navigating the surgical instruments. The Magellan™(Hansen Medical, Mountain View, CA, USA) robotic system, specialised for peripheral vascular procedures, was used with the 6Fr Magellan steerable robotic catheter with a 9.5Fr sheath. This catheter is capable of $180^{\circ }$ multidirectional articulation while the sheath adds $90^{\circ }$ articulation. The operator controls the robot and navigates the catheter remotely from a workstation using a joystick device, with the aid of fluoroscopy imaging and orientation information superimposed in the fluoroscopy screen. Standard endovascular instruments and devices (balloons, stents) can also be percutaneously inserted and navigated with the Magellan system. In our set-up, the robotic arm was placed on the top of the surgical table to access the phantom model while the surgeon operated from the control console, placed nearby.

### Participants

Twelve endovascular surgeons agreed to participate in this study. Participants were categorised into two groups based on their previous endovascular experience. The two groups were labelled as: novice group ($n=6$) with no prior experience in endovascular interventions and the expert group, comprising of individuals with more than 100 endovascular operations in their career. No individual had extensive prior experience with the robotic system and no test runs took place before the execution of the experiments. Each participant was briefed on the study and asked to perform two executions: one with the conventional equipment and one with the Magellan platform in random order.

**Fig. 3:**
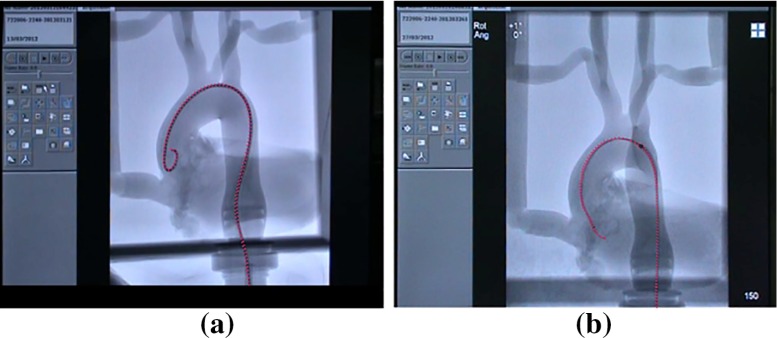
Annotation of the shape of the catheter, while inserted in the phantom, in fluoroscopy images; **a** conventional catheter annotated at the end of stage 1; **b** robotic catheter annotated during stage 2

## Data processing

The 2D location, in pixel coordinates, of the catheter/guidewire tip in the fluoroscopic image was extracted, on a frame-by-frame basis, using a semi-automated tip tracking algorithm. The semi-automatic software allows frame-by-frame video seeking with user interaction at each frame allowing the definition of points and contours over the video. The software embeds tracking of points using the Lucas–Kanade algorithm or by brute force, normalised cross-correlation matching [21, 22]. User interaction is required to bootstrap the tracking at points of failure or drift because of fast catheter motions, blur or poor contrast. The output of the tracking algorithm is the catheter tip motion. In the video recordings, the temporal limits of the two TAVI stages were manually annotated and the total procedure time for each stage was extracted. The fluoroscopy screen was recorded during the experiments, and the video sequences were analysed. A pre-processing step was required because the fluoroscopy monitor was captured with an external camera from an unfixed position thus contained some degree of screen view bias. In all recordings, the entire fluoroscopy screen was visible which allowed us to shear the screens from different videos and using affine transformations, warp them to the same size ($720\times 576$). This step rectified the perspective distortion and normalised the obtained trajectory coordinates from each recording.

In order to evaluate the safe navigation of the catheter in a quantitative way, we elected to investigate the tip’s trajectory with respect to its distance from the vessel wall. The first step was to segment the shape and geometry of the vasculature in the fluoroscopic image. This was done manually to avoid segmentation inaccuracies, by isolating the portion of the image that contains the aorta model and transforming it into a greyscale image. We then extracted the shape of the phantom by converting this image into a binary one using a cut-off threshold value. Pixels with greyscale value above the threshold were assigned value “1” while the ones below, the value “0”. In the obtained binary image, the aorta model is delimitated by the white pixels. Finally, we calculate the distance of each white (“1”) pixel that corresponds to a track of the catheter’s trajectory to the nearest black (“0”) pixel, which in essence defines the edge of the vessel wall. This allows us to calculate features like the average distance of the tip’s locations to the nearest point of the vessel wall. Our main hypothesis is that experienced interventionists should be able to maintain a relatively large distance from the vessel wall, thus minimising the danger for potential tissue damage. To augment our dataset for this analysis, the shape of the catheter was manually annotated, through crowdsourcing in a subset of frames from each recorded sequences. Annotation was carried out in Amazon Mechanical Turk with an HTML-/JavaScript-based contour annotation tool tuned for catheter-like shapes [1]. The catheter-shape annotations were normalised in a similar way as the tracked coordinates and then interpolated using spline interpolation so that the catheter was annotated with the same number of points in every frame. Figure 3 shows two examples of catheter-shape annotation.

**Fig. 4:**
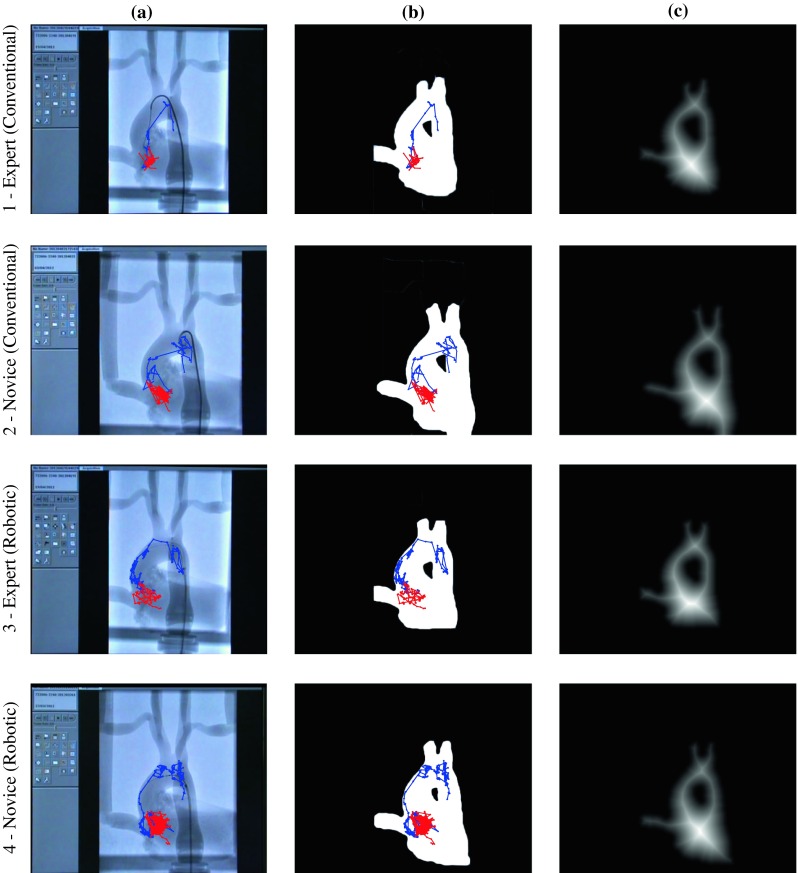
Applied image processing steps; Column **a** original frames of the fluoroscopy monitor with tip’s positions (*blue* stage 1, *red* stage 2); Column **b** binary image with segmented aorta model; Column **c** distance image showing the distance of *each pixel* of the phantom model (*white pixels*) from the closest point on the vessel wall (*first black pixel*). Intensity of *white colour* denotes higher distance value. Comparing **1**, **b** and **2**, **b** we note the more efficient handling of the catheter by the expert, particularly in stage 1 (*blue line*)

Figure 4 illustrates the three processing steps we followed (catheter/guidewire tip tracking, model segmentation, distance calculation) in representative experimental executions from an expert (rows 1 and 3) and a novice participant (rows 2 and 4) with both the conventional catheter (rows 1 and 2) and the robotic system (rows 3 and 4). The higher efficiency of the expert surgeon, in terms of time and movement, is again evident by the trajectory of the tip particularly during the first stage. It is also clear that the two operators required more steps to complete the procedure when using the robotic catheter.

**Table 1:** Median values and *p* values of the kinematic features

Operators	Novices	Experts	*p* value (MW)
Procedure time (s)—stage 1			
Conventional	239.1	34.9	*0.008*
Robotic	200.8	149.6	0.309
*p* value (Wi)	0.687	*0.031*	
Procedure time (s)—stage 2			
Conventional	208	111.2	0.240
Robotic	350.3	332.0	0.699
*p* value (Wi)	0.312	0.562	
Average speed (px/s)—stage 1			
Conventional	10.5	22.3	0.132
Robotic	7.1	8.0	0.699
*p* value (Wi)	0.312	*0.031*	
Average speed (px/s)—stage 2			
Conventional	12.5	22	0.393
Robotic	6.7	8.2	0.818
*p* value (Wi)	*0.031*	0.093	
Average acceleration (px/s^2^)—Stage 1			
Conventional	62	67.4	0.937
Robotic	28.6	32.7	0.699
*p* value (Wi)	0.062	*0.031*	
Average acceleration (px/s^2^)—Stage 2			
Conventional	50.2	77.9	0.240
Robotic	32.1	43.4	0.393
*p* value (Wi)	0.062	*0.031*	
Dimensionless jerk—stage 1			
Conventional	592.3	0.33	*0.008*
Robotic	516.2	226.7	0.393
*p* value (Wi)	0.843	*0.031*	
Dimensionless jerk—stage 2			
Conventional	667.7	38.2	0.484
Robotic	5392.9	3481.2	0.699
*p* value (Wi)	0.312	0.562	
Tip average distance to wall (px)—stage 1			
Conventional	23.0	27.1	0.930
Robotic	24.7	24.9	0.818
*p* value (Wi)	1	0.625	
Tip average distance to wall (px)—stage 2			
Conventional	42.7	27.7	0.246
Robotic	47.0	39.5	0.588
*p* value (Wi)	0.437	0.187	
Shape average distance to wall (px)—stage 1			
Conventional	24.2	24.3	0.536
Robotic	25.6	29.1	0.484
*p* value (Wi)	0.562	0.312	
Shape average distance to wall (px)—stage 2			
Conventional	19.5	17.9	0.536
Robotic	20.2	20.1	0.937
*p* value (Wi)	0.437	0.312	

Statistically significant values are highlighted in italics

From the manual annotation of the two stages of the experiment, the total procedure time was extracted for each one. Motion-based metrics were calculated from the tracks of the catheter’s path. During the experiments, we noticed that occasionally the instrument would remain stationary for a period of time. We therefore elected to calculate the average speed (calculated as $v_\mathrm{d} = \hbox {PL}/T_\mathrm{p}$, $T_\mathrm{p}$: Procedure time, PL: total path length) as an indication of movement efficiency as the path length alone would not be indicative of these pauses. Additional features that were calculated include the average magnitude of the acceleration vector (calculated as the 2-norm of the acceleration components in the two axes: $a_\mathrm{d}=\sqrt{a_x^2+a_y^2}$) and the dimensionless jerk. Dimensionless jerk is a measure used to describe smoothness in the shape of a movement and is defined in such a way to be independent of duration and amplitude [6]. Considering 2D motion it is given as:

1 $$\begin{aligned} j_\mathrm{d} = \left( 0.5 \int _{t_i}^{t_e}{(\dddot{x}(t))^2 + (\dddot{y}(t))^2 \hbox {d}t} \right) \cdot \dfrac{T_\mathrm{p}^5}{\hbox {PL}^2} \end{aligned}$$

The catheter’s trajectory coordinates were also used to calculate the average distance (measured in pixels) to the nearest vessel wall point from the derived distance images.

## Results and discussion

### Statistical analysis

**Fig. 5:**
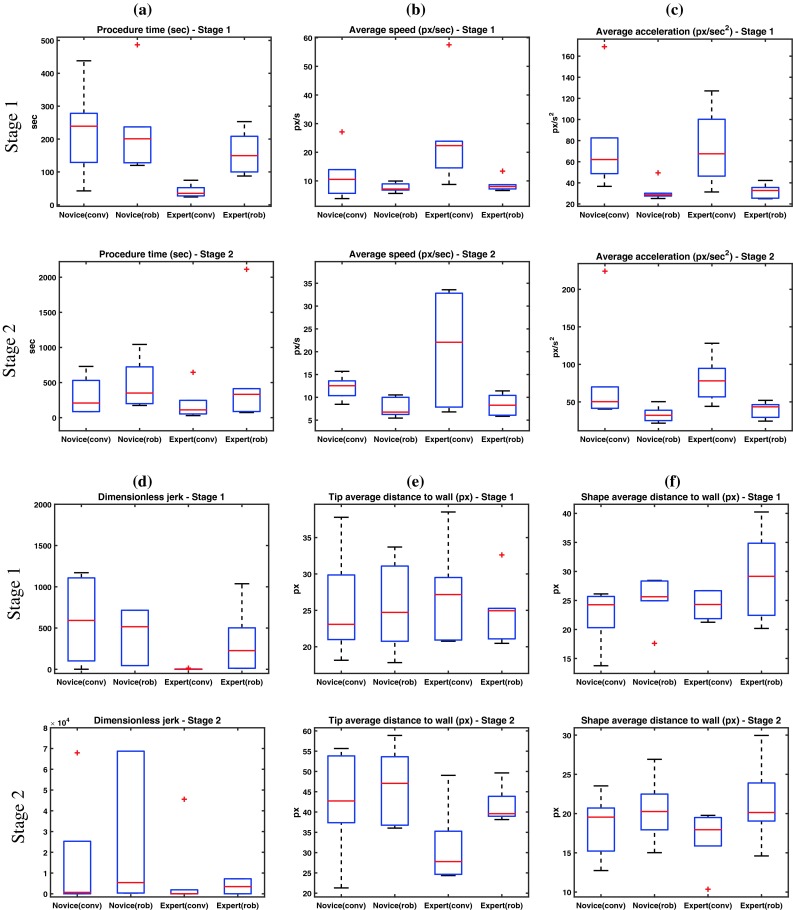
*Box* plots of the derived features for both stages: **a** procedure time, **b** average speed, **c** average acceleration, **d** dimensionless jerk, **e** average tip distance to the wall, **f** average shape distance to the wall

Results between the two experience groups were compared using the Mann–Whitney *U* test (MW) while to compare the two types of catheters in each experience group we used the Wilcoxon signed-rank test (Wi). Statistical significance was considered for *p* value $<$0.05. Median values for procedure time (s), average speed (px/s), average acceleration magnitude (px/s^2^), dimensionless jerk and average distance to vessel wall (both for the catheter’s tip and shape) are listed in Table 1. The box plots of the six features are illustrated in Fig. 5, with the box edges set to the 25th and the 75th percentile. We note that outliers are limited in our dataset.

From Table 1, we observe that the median value of the procedure time is characteristically different among the two groups. Expert surgeons complete both stages faster (34.9 vs 239.1 s, $p=0.008$) and stage 2 (111.2 vs 208 s) than novices, with the conventional surgical equipment used. This observation attests to the experts refined skills in completing tasks faster and is consistent with previous studies on endovascular simulator skills that have reported similar high correlation of the total procedure time to the level of expertise [8, 10]. The *p* value (0.008) shows statistical significance in stage 1. The robotic system generally results in higher completion times. Only the novice group in stage 1 competes the task faster with the robot (200.8 vs 239.1 s). In all other circumstances, the procedure times increase, while the difference is significant in the experts group during stage 1 ($p=0.031$). The increase can be attributed to the fact that participants had no operational familiarity with the robotic system.

Average speed and average acceleration demonstrate a similar trend, with experts achieving higher values. The difference is higher when conventional equipment is used, where expert operators exhibited a higher average speed than novices in both stages, 22.3 versus 10.5 px/s in stage 1 and 22 versus 12.5 px/s in stage 2, and higher average acceleration, 67.4 versus 62 and 77.9 versus 50.2 px/s^2^ in stage 1 and stage 2, respectively. Considerable lower and fairly similar, with experts being marginally faster, speed and acceleration median values were achieved by the two groups with the robotic system. The difference among the two surgical systems shows statistical importance for the average speed in stage 1 of the experts , the average speed in stage 2 of the novices and the average acceleration in stage 1 and stage 2 of the experts with a value of $p=0.031$. Experts demonstrated smaller median values of dimensionless jerk than novices in both stages with both catheter types, with the difference being statistically important ($p=0.008$), in stage 1 with conventional equipment. This observation, of a metric independent of duration and amplitude, provides evidence of the experts refined skills to navigate the catheter in a smooth and efficient way.

Although statistical significance is not always observed, there is a consistent trend, in both stages and particularly with standard catheters used in everyday surgical practice, of lower median values for procedural time and dimensionless jerk and higher for average speed and average acceleration by the expert group. This suggests that highly skilled interventionalists are able to complete TAVI cannulation tasks in a faster, more efficient manner without unnecessary movements, compared to novices that are still under training and developing their dexterous manipulations skills. The above conclusion justifies our main hypothesis that motion-based metrics can be indicative of surgical experience.

The median values of the average distance to the vessel wall are also listed in Table 1. Both groups demonstrate similar performance in stage 1 with both catheter types. On the other hand, in stage 2 which involves crossing the aortic valve and navigating the catheter into the left ventricle, a higher average distance was exhibited in both groups when using the robotic catheter. A similar trend is observed when the catheter-shape annotations are used. Again median values are close but slightly higher with the robotic system. Although the *p* values do not reveal statistical significance, this observation may be indicative of the robotic system ability for more precise and safe navigation away from calcification deposits, thus limiting potential danger for vessel damage and embolisation.

**Fig. 6:**
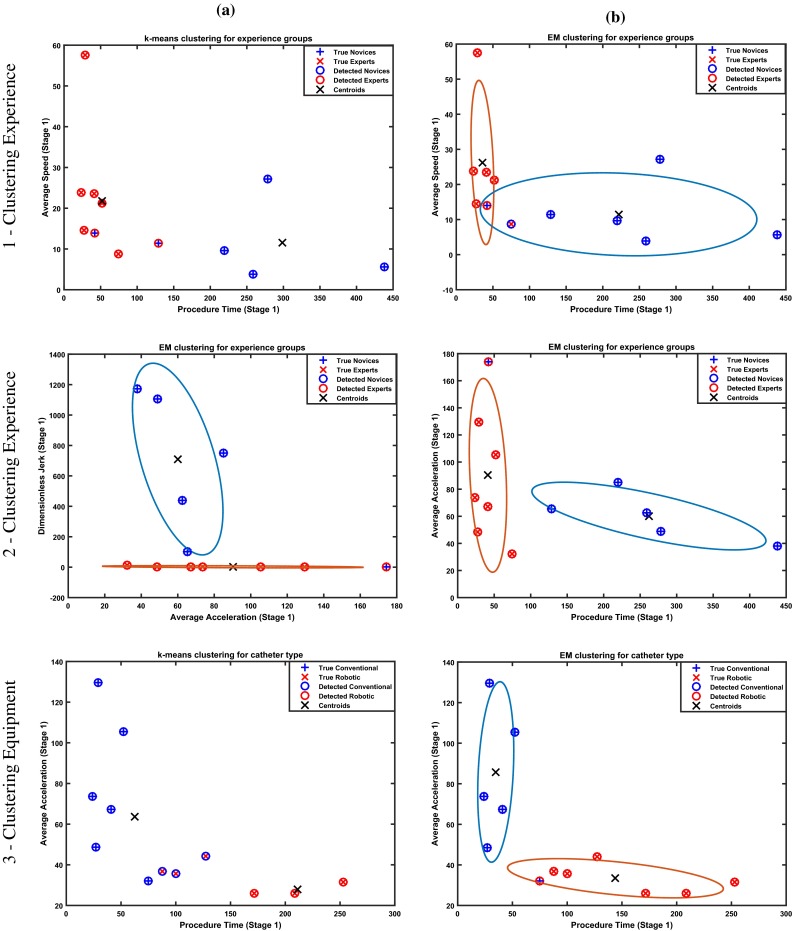
Clustering results for the experience groups (**1**, **2**) and the catheter type (**3**) in the experts group, using combinations of the $T_\mathrm{p}$, $v_\mathrm{d}$, $a_\mathrm{d}$, $j_\mathrm{d}$—**1**, **a** k-means (10/12—83 %); **1**, **b** EM (10/12—83 %); **2**, **a** EM (11/12—91 %); **2**, **b** EM (11/12 - 91 %); **3, a** k-means (9/12—75 %); **3**, **b** EM (11/12—91 %); the cluster centroids are given for both algorithms and the 70 % confidence interval is depicted in the EM cases

### Clustering analysis

To evaluate the ability for primarily discriminating experience groups but also catheter equipment, the derived feature sets were used as inputs in clustering methods. Combining autonomously generated metrics with intelligent algorithms and ultimately designing a system to objectively assess and categorise surgical performance will find direct application in the training process of new surgeons where valuable feedback can guide trainees to develop important dexterity skills without expert supervision. Such systems can also be used for evaluating the clinical translation of new technology. Additionally, they will facilitate the design of intelligent surgical robot systems, capable of performing with some level of autonomy. Two algorithms are investigated here: k-means clustering and expectation maximisation (EM). k-means clustering attempts to separate a collection of observations into groups in such a way that the sum of the distances of the points of each group to the cluster centre (within clusters sum of squares) is minimised. The goal of EM clustering is to estimate the parameters (mean and standard deviations) of a probability model (Gaussian mixture) that describes each cluster by maximising the likelihood of the observations.

Given the results of the statistical analysis, we elected to use the features that demonstrated statistical significance (or where close) for classification. Specifically procedure time ($T_\mathrm{p}$), average speed ($v_\mathrm{d}$), average acceleration ($a_\mathrm{d}$) and dimensionless jerk ($j_\mathrm{d}$) in phase 1 were used for classifying the participants of the two experience groups and the procedure time and the average acceleration (in the experts group) to classify the different surgical equipment. The only other input was the number of clusters ($n=2$) that have to be formulated. Both algorithms were initialised randomly. The obtained classification results for both algorithms are illustrated in Fig. 6. Maximum obtained accuracy was 83 % (10/12) and 91 % (11/12) for k-means and EM, respectively, in classifying surgical experience and 75 % (9/12) for k-means and 91 % (11/12) for EM, in the classification of the equipment with different combinations of the feature set.

## Conclusions

This paper presented an investigation on deriving and validating objective measures for endovascular surgical skills assessment, based on analysing the motion and manipulation patterns of surgical tools. A cohort of 12 participants separated into two groups (novices, experts) performed two stages of a TAVI operation on a phantom silicon model, with conventional surgical tools and the Magellan endovascular robotic platform. Video sequences of the fluoroscopy screen were recorded and used to track the 2D position of the catheter/guidewire tip as well as to segment the aorta model. The motion-based metrics that were investigated are: total procedure time, average speed, average acceleration magnitude, dimensionless jerk and average distance (of the catheter’s tip and shape) to the vessel wall, a metric we believe is indicative of safer catheter navigation. As expected, the two groups demonstrated different procedure times especially in the arch navigation stage ($p=0.008$), with experts completing the tasks faster than novices. Moreover, expert surgeons exhibited higher median values in average speed and acceleration in both stages. Movement smoothness was evaluated with the dimensionless jerk, a metric designed to be independent of duration and amplitude, in which experts, as expected, demonstrated smaller median values with the difference being statistically important ($p=0.008$) in stage 1. Experiments with the Magellan platform resulted in higher value for procedure times and dimensionless jerk and lower speed and acceleration values. This can be attributed to the robot motion scaling for fine manipulation and potentially to the participants’ lack of experience in operating the robotic system. The discrimination potential of the derived motion-based metrics was evaluated with unsupervised clustering experiments. Two established algorithms were employed (k-means, EM) and achieved 83 % (k-mean)–91 % (EM) accuracy in classifying the participants to their respective experience group and 75 % (k-means), 91 % (EM) in discriminating among the two types of equipment used. The obtained high level of accuracy validates our basic hypothesis that motion-based analysis can indeed provide skill-representative metrics. Inspired by the criteria evaluated in traditional surgical assessment methods (GRS, checklists), this work introduced the distance to the vessel wall as a metric to evaluate safe catheter navigation. Although the average distance to the vessel wall did not demonstrate statistical significance among the two groups, it was found to be higher during stage 2 which involves the navigation of the catheter through the aortic valve. Based on this, the robotic system appears to facilitate safer catheter manipulation.

Future work will focus on the kinematic analysis of the entire catheter, instead of just the tip, as well as on modelling surgical execution using the kinematic features in order to assess performance more thoroughly besides producing a classification result. In addition, we target at fully automating the catheter tracking algorithm and phantom segmentation, currently semi-automated, so that minimum user interaction is required.
